# Benign Intracerebral Hemorrhage: A Population at Low Risk for Hematoma Growth and Poor Outcome

**DOI:** 10.1161/JAHA.118.011892

**Published:** 2019-04-11

**Authors:** Qi Li, Wen‐Song Yang, Yi‐Qing Shen, Xiong‐Fei Xie, Rui Li, Lan Deng, Ting‐Ting Yang, Fa‐Jin Lv, Fu‐Rong Lv, Guo‐Feng Wu, Zhou‐Ping Tang, Joshua N. Goldstein, Peng Xie

**Affiliations:** ^1^ Department of Neurology The First Affiliated Hospital of Chongqing Medical University Chongqing China; ^2^ Department of Radiology The First Affiliated Hospital of Chongqing Medical University Chongqing China; ^3^ Emergency Department The Affiliated Hospital of Guizhou Medical University Guiyang China; ^4^ Department of Neurology Tongji Hospital Tongji Medical College Huazhong University of Science and Technology Wuhan China; ^5^ Division of Neurocritical Care and Emergency Neurology Massachusetts General Hospital Harvard Medical School Boston MA

**Keywords:** computed tomography, intracerebral hemorrhage, neuroimaging, outcome, stroke, Cerebrovascular Disease/Stroke

## Abstract

**Background:**

To define benign intracerebral hemorrhage (ICH) and to investigate the association between benign ICH, hematoma expansion, and functional outcome.

**Methods and Results:**

We analyzed a prospectively collected cohort of patients with ICH, who presented within 6 hours of symptom onset between July 2011 and February 2017 to a tertiary teaching hospital. Follow‐up computed tomographic scanning was performed within 36 hours after initial computed tomographic scanning. Benign ICH was operationally defined as homogeneous and regularly shaped small ICH. The presence of benign ICH was judged by 2 independent reviewers (Q.L., W.Y.) on the basis of the admission computed tomographic scan. Functional independence was defined as a modified Rankin Scale score of 0 to 2 at 3 months. The associations between benign ICH, hematoma expansion, and functional outcome were assessed by using multivariable logistic regression analyses. A total of 288 patients with ICH were included. Benign ICH was found in 48 patients (16.7%). None of the patients with benign ICH had early hematoma expansion. The sensitivity, specificity, positive predictive value, negative predictive value, and accuracy of benign ICH for predicting functional independence at 3 months were 30.7%, 96.6%, 90.0%, 60.0%, and 0.637, respectively.

**Conclusions:**

Patients with benign ICH are at low risk of hematoma expansion and poor outcome. These patients may be safe for less intensive monitoring and are unlikely to benefit from therapies aimed at preventing ICH expansion.

**Clinical Perspective**

**What Is New?**

- In this prospective cohort study, we have proposed a concept of benign intracerebral hemorrhage (ICH) to define a population at low risk for hematoma growth and poor outcome.
- Patients with benign ICH are less likely to have early hematoma expansion. The presence of benign ICH was associated with functional independence.

**What Are the Clinical Implications?**

- Benign ICH is unlikely to expand and should be excluded from antiexpansion studies.
- In limited resource settings, patients with benign ICH may require less need for intensive monitoring.

## Introduction

Intracerebral hemorrhage (ICH) is a major public health burden that accounts for ≈2 million strokes worldwide.^1^, ^2^ Mortality is high in the short‐term phase, and less than half of survivors achieve functional independence.^3^ Hematoma growth has been observed in approximately one third of patients presenting within the first hours and is associated with poor functional outcome.^4^, ^5^, ^6^, ^7^, ^8^ Therefore, identifying which patients will expand (and which will not) is critical in guiding care.

Several factors are known to be associated with risk of expansion and/or poor outcome, including baseline hematoma volume, age, presence of intraventricular hemorrhage, and infratentorial location.^6^, ^7^, ^9^ Moreover, early time to presentation may mark those early in the disease course and at highest risk.^10^, ^11^, ^12^

Neuroimaging can also be used to identify those at high risk for expansion and poor functional outcome; findings, including the blend sign, black hole sign, satellite sign, computed tomographic (CT) hypodensities, and island sign, all predict further hemorrhage and are associated with poor outcome.^13^, ^14^, ^15^, ^16^, ^17^, ^18^, ^19^ However, the converse has not yet been well studied (ie, how to identify those at such low risk for expansion that they be excluded from trials of antiexpansion therapies). We defined a group of regularly shaped and homogeneous small hematomas as “benign ICH.” The aim of our study was to investigate whether some patients with ICH may have a benign course and to explore the prevalence, clinical characteristics, and association of benign ICH with hematoma growth and functional outcome.

## Methods

The data that support the findings of this study are available from the corresponding authors on reasonable request.

### Study Population

We prospectively included patients presenting with acute primary ICH to our hospital between July 2011 and February 2017. Patients were included in our analysis if they underwent the following: (1) a baseline CT scan within 6 hours after onset of symptoms and (2) a follow‐up CT scan within 36 hours after the initial CT scan. Patients were excluded if they had primary intraventricular hemorrhage, anticoagulant‐associated bleeding, secondary ICH attributable to arteriovenous malformation or intracranial aneurysm, or surgery before follow‐up CT, or if they were lost to follow‐up at 3 months.

### Clinical Data Collection and Outcome Assessment

Trained neurologists obtained and recorded the baseline demographic and clinical data, which included age, sex, medical history, and medication use history. Other data captured included admission blood pressure, ICH score, Glasgow Coma Scale (GCS) score, National Institutes of Health Stroke Scale (NIHSS) score, premorbid modified Rankin Scale (mRS) score, the time from symptom onset to baseline CT scanning, and the time interval between baseline CT and follow‐up CT scanning. The study investigators promised to provide free medical consultation during the study period for patients who were willing to participate in our study. Clinical outcomes were assessed using the mRS score through a telephone interview by trained medical staff at the 3‐month follow‐up. Good outcome was defined as a 3‐month mRS score of 0 to 3, and poor outcome was defined as a 3‐month mRS score of 4 to 6. Last, functional independence was defined as an mRS score of 0 to 2.^20^, ^21^

### Imaging Interpretation and Analysis

Noncontrast CT scans were performed, as previously described.^14^, ^15^, ^16^ The CT images were saved in DICOM format for further review. Trained neurologists (Q.L., W.Y.) who were blinded to the clinical and outcome data independently reviewed all CT images. Discrepancies between the readers were settled by joint discussion until consensus was reached. The hematoma volume was measured using the ABC/2 method, as described previously.^22^ Early hematoma expansion was defined as an increase in hematoma volume of >6 mL or 33% between baseline and follow‐up CT scanning.^23^

### Definition of Small ICH and Benign ICH

**Figure 1:**
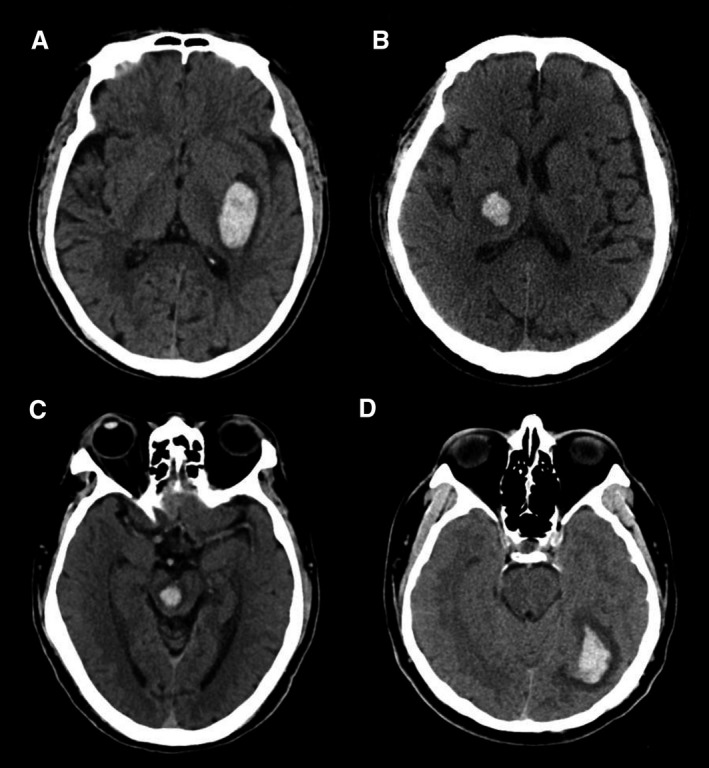
Representative images of benign intracerebral hemorrhage (ICH). Representative axial noncontrast computed tomographic images in patients with benign ICH. Basal ganglia (**A**), thalamic (**B**), brainstem (**C**), and lobar benign (**D**) hemorrhages. Benign hematomas are relatively regular in shape and homogeneous in density.

**Figure 2:**
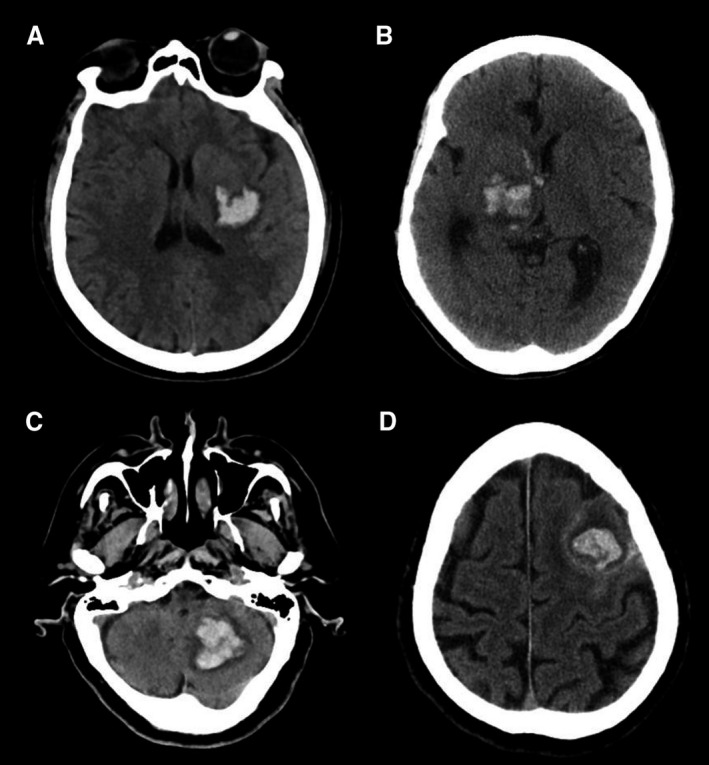
Illustration of benign intracerebral hemorrhage (ICH) mimics. Representative images of hematomas that mimic benign ICH. **A**, A homogeneous basal ganglia hemorrhage. The hematoma is irregularly shaped. **B**, A heterogeneous and irregular thalamic hematoma with intraventricular extension. **C**, A heterogeneous cerebellar hemorrhage. **D**, A heterogeneous lobar hematoma with subarachnoid hemorrhage.

We operationally defined a “small ICH” on the basis of the following criteria: (1) if brainstem hemorrhage, volume <3 mL; (2) if cerebellar hemorrhage, volume <5 mL; (3) if thalamic hemorrhage, volume <10 mL; (4) if basal ganglia hemorrhage, volume <10 mL; and (5) if lobar hemorrhage, volume <15 mL. We then operationally defined benign ICH using the following criteria: (1) a small ICH, as above; (2) no concurrent intraventricular hemorrhage or subarachnoid hemorrhage; (3) a homogeneous and regularly shaped hematoma; and (4) no evidence of blend sign, black hole sign, CT hypodensities, island sign, or satellite sign.^13^, ^14^, ^15^, ^16^, ^17^, ^18^, ^19^ Benign ICH, as defined herein, and its mimics are illustrated in Figures 1 and 2, respectively. Malignant small ICH is defined as one that meets criteria for small ICH but not benign ICH.

### Statistical Analysis

Statistical analyses were performed using SPSS 19.0 software. Categorical variables are presented as percentages. Continuous variables are presented as mean (SD) if normally distributed or median (interquartile range [IQR]) if not normally distributed. The between‐group differences were assessed using a χ^2^ test, a Fisher exact test, a Student *t* test, or a Mann‐Whitney *U* test, as appropriate. The interrater reliability for defining benign ICH was calculated using κ statistics. We calculated the sensitivity, specificity, positive predictive value, negative predictive value, and accuracy of benign ICH to predict functional independence at 3 months. The threshold of significance was set at *P*<0.05.

### Standard Protocol Approval, Registration, and Patient Consent

All study procedures and protocols involving human participants were conducted in accordance with the ethical standards of the 1964 Declaration of Helsinki, and the study was approved by the Ethics Committee of the First Affiliated Hospital of Chongqing Medical University (Chongqing, China). Written informed consent was obtained from all participants or their legally authorized representatives before participation.

## Results

During the study period, a total of 370 patients met the inclusion criteria. After applying exclusion criteria, 77 patients were excluded. A total of 5 patients refused to participate in our study, leaving 288 for final analysis (Figure S1). None of the included patients were transferred in from other institutions. A total of 21 patients (7.3%) had withdrawal of life‐sustaining treatment in our study after enrollment. The mean age of the participants was 61 years (range, 27–94 years), and 191 patients (66.3%) were men. The median time from symptom onset to baseline CT scan was 2 hours (IQR, 1–4 hours). Small ICH occurred in 108 patients (37.5%). Patients with small ICH had a lower admission NIHSS score (median, 7.5 [IQR, 3–12] versus 13 [IQR, 8–24]; *P*<0.001), a higher GCS score (median, 15 [IQR, 14–15] versus 13 [IQR, 9–14]; *P*<0.001), a lower ICH score (median, 0 [IQR, 0–1] versus 1 [IQR, 0–2]; *P*<0.001), and lower rates of early hematoma expansion (15.7% versus 40.6%; *P*<0.001) than those without small ICH (Table 1).

**Table 1:** Comparison of Baseline Demographic, Clinical, and Radiological Characteristics Between Patients With and Without Small and Benign ICH

Variables	Patients					
	With Small ICH (n=108, 37.5%)	Without Small ICH (n=180, 62.5%)	*P* Value	With Benign ICH (n=48, 16.7%)	Without Benign ICH (n=240, 83.3%)	*P* Value
Demographic						
Age, mean (SD), y	62.1 (12.5)	58.9 (11.7)	0.030^a^	60.5 (12.9)	60.0 (12.0)	0.774
Male sex, n (%)	67 (62.0)	124 (68.9)	0.234	31 (64.6)	160 (66.7)	0.780
Medical history, n (%)						
Alcohol consumption	43 (39.8)	85 (47.2)	0.221	22 (45.8)	106 (44.2)	0.832
Smoking	48 (44.4)	89 (49.4)	0.411	25 (52.1)	112 (46.7)	0.493
Hypertension	79 (73.1)	123 (68.3)	0.387	31 (64.6)	171 (71.3)	0.357
Diabetes mellitus	13 (12.0)	17 (9.4)	0.486	3 (6.3)	27 (11.3)	0.301
Clinical features						
Systolic blood pressure, mean (SD), mm Hg	168.9 (24.9)	171.2 (30.6)	0.516	165.0 (23.3)	171.4 (29.5)	0.161
Diastolic blood pressure, mean (SD), mm Hg	97.3 (16.2)	100.0 (18.5)	0.207	98.4 (16.0)	99.1 (18.0)	0.797
Admission GCS score, median (IQR)	15 (14–15)	13 (9–14)	<0.001^a^	15 (14–15)	13 (9–14)	<0.001^a^
Admission NIHSS score, median (IQR)	7.5 (3–12)	13 (8–24)	<0.001^a^	5.5 (2–10)	12 (7–20)	<0.001^a^
Baseline ICH volume, median (IQR), mL	6.5 (4.1–8.8)	18.8 (14.0–30.6)	<0.001^a^	5.7 (2.4–8.4)	15.2 (9.9–24.8)	<0.001^a^
IVH at baseline CT, n (%)	31 (28.7)	62 (34.4)	0.313	0 (0)	93 (38.8)	<0.001^a^
Time from onset to CT, median (IQR), h	2 (1–4)	2 (1–3)	0.139	3 (1–5)	2 (1–3)	0.075
SAH at baseline CT, n (%)	4 (3.7)	29 (16.1)	0.001^a^	0 (0)	33 (13.8)	0.006
Hematoma growth, n (%)	17 (15.7)	73 (40.6)	<0.001^a^	0 (0.0)	90 (37.5)	<0.001^a^
ICH score, median (IQR)	0 (0–1)	1 (0–2)	<0.001^a^	0 (0)	1 (0–2)	<0.001^a^
Speed of uHG, median (IQR), mL/h	2.7 (1.3–5.0)	11.3 (5.8–21.7)	<0.001^a^	2.2 (1.1–4.1)	8.6 (4.1–17.9)	<0.001^a^
Withdrawal of life‐sustaining treatment, n (%)	0 (0)	21 (8.8)	0.068^a^	1 (0.9)	20 (11.1)	0.001^a^
ICH locations, n (%)						
Lobar hemorrhage	12 (11.1)	23 (12.8)	0.675	6 (12.5)	29 (12.1)	0.936
Basal ganglia hemorrhage	50 (46.3)	112 (62.2)	0.008^a^	28 (58.3)	134 (55.8)	0.750
Thalamic hemorrhage	35 (32.4)	35 (19.4)	0.013^a^	9 (18.8)	61 (25.4)	0.326
Brainstem hemorrhage	5 (4.6)	4 (2.2)	0.431	3 (6.3)	6 (2.5)	0.363
Cerebellar hemorrhage	6 (5.6)	6 (3.3)	0.542	2 (4.2)	10 (4.2)	1.000
Outcome						
In‐hospital mortality, n (%)	4 (3.7)	13 (7.2)	0.220	0 (0)	17 (7.1)	0.117
90‐d Mortality, n (%)	9 (8.3)	46 (25.6)	<0.001^a^	0 (0)	55 (22.9)	<0.001^a^
90‐d mRS score, median (IQR)	2 (1–3)	4 (1–6)	<0.001^a^	1 (0–2)	3 (1–5)	<0.001^a^
90‐d mRS score of 0–2, n (%)	67 (62.0)	73 (40.6)	<0.001^a^	43 (89.6)	97 (40.4)	<0.001^a^

CT indicates computed tomography; GCS, Glasgow Coma Scale; ICH, intracerebral hemorrhage; IQR, interquartile range; IVH, intraventricular hemorrhage; mRS, modified Rankin Scale; NIHSS, National Institutes of Health Stroke Scale; SAH, subarachnoid hemorrhage; uHG, ultraearly hematoma growth.

a Indicates *P* value <0.05.

Of the 288 patients with ICH, 48 (16.7%) had benign ICH. The interrater agreement was excellent for judging benign ICH (κ, 0.88; 95% CI, 0.80–0.95). Patients with benign ICH had smaller baseline hematoma volumes (median, 5.7 [IQR, 2.4–8.4] mL versus 15.2 [9.9–24.8] mL; *P*<0.001), lower NIHSS scores (median, 5.5 [IQR, 2–10] versus 12 [IQR, 8–20]; *P*<0.001), lower ICH scores (median, 0 [IQR, 0] versus 1 [IQR, 0–2]; *P*<0.001), and higher GCS scores (median, 15 [14–15] versus 13 [IQR, 9–14]; *P*<0.001) (Table 1). The rate of hematoma growth was significantly lower in patients with benign ICH than in those without benign ICH (0% versus 37.5%; *P*<0.001; RR (relative risk), 0.63; 95% CI, 0.57–0.69). Of the 108 patients with small ICH, 60 (55.6%) had malignant small ICH. Table 2 compares those with benign ICH with those with malignant but small ICH. Notably, patients with benign ICH had lower NIHSS scores (median, 5.5 [IQR, 2–10] versus 10 [IQR, 4–15]; *P*=0.004), had higher GCS scores (median, 15 [IQR, 14–15] versus 14 [IQR, 13–15]; *P*=0.001), and are less likely to expand (0% versus 28.3%, *P*<0.001) compared with patients with malignant small ICH. In addition, the incidence of functional independence (mRS score, 0–2) was higher in patients with benign ICH than in those with malignant small ICH (89.6% versus 40.0%; *P*<0.001).

**Table 2:** Comparison of Baseline Demographic, Clinical, and Radiological Characteristics Between Patients With Benign ICH and Malignant Small ICH

Variables	Patients With Benign ICH (n=48, 44.4%)	Patients With Malignant Small ICH (n=60, 55.6%)	*P* Value
Demographic			
Age, mean (SD), y	60.5 (12.9)	63.3 (12.2)	0.254
Male sex, n (%)	31 (64.6)	36 (60.0)	0.626
Medical history, n (%)			
Alcohol consumption	22 (45.8)	21 (35.0)	0.253
Smoking	25 (52.1)	23 (38.3)	0.153
Hypertension	31 (64.6)	48 (80.0)	0.072
Diabetes mellitus	3 (6.3)	10 (16.7)	0.098
Clinical features			
Systolic blood pressure, mean (SD), mm Hg	165.0 (23.3)	172.0 (25.8)	0.149
Diastolic blood pressure, mean (SD), mm Hg	98.4 (16.0)	96.4 (16.4)	0.531
Admission GCS score, median (IQR)	15 (14–15)	14 (13–15)	0.001^a^
Admission NIHSS score, median (IQR)	5.5 (2–10)	10 (4–15)	0.004^a^
Baseline ICH volume, median (IQR), mL	5.7 (2.4–8.4)	6.7 (4.7–9.0)	0.053
IVH at baseline CT, n (%)	0 (0)	31 (51.7)	<0.001^a^
Time from onset to CT, median (IQR), h	3 (1–5)	2 (1–3.88)	0.236
SAH at baseline CT, n (%)	0 (0)	4 (6.7)	0.190
Hematoma growth, n (%)	0 (0)	17 (28.3)	<0.001^a^
ICH score, median (IQR)	0 (0)	1 (0–1)	<0.001^a^
Speed of uHG, median (IQR), mL/h	2.2 (1.1–4.1)	3.4 (1.6–6.6)	0.012^a^
ICH locations, n (%)			
Lobar hemorrhage	6 (12.5)	6 (10.0)	0.681
Basal ganglia hemorrhage	28 (58.3)	22 (36.7)	0.025^a^
Thalamic hemorrhage	9 (18.8)	26 (43.3)	0.007^a^
Brainstem hemorrhage	3 (6.3)	2 (3.3)	0.798
Cerebellar hemorrhage	2 (4.2)	4 (6.7)	0.888
Outcome			
In‐hospital mortality, n (%)	0 (0)	4 (6.7)	0.190
90‐d Mortality, n (%)	0 (0)	9 (15.0)	0.014^a^
90‐d mRS score, median (IQR)	1 (0–2)	3 (1–4)	<0.001^a^
90‐d mRS score of 0–2, n (%)	43 (89.6)	24 (40.0)	<0.001^a^

CT indicates computed tomography; GCS, Glasgow Coma Scale; ICH, intracerebral hemorrhage; IQR, interquartile range; IVH, intraventricular hemorrhage; mRS, modified Rankin Scale; NIHSS, National Institutes of Health Stroke Scale; SAH, subarachnoid hemorrhage; uHG, ultraearly hematoma growth.

a Indicates *P* value <0.05.

**Figure 3:**
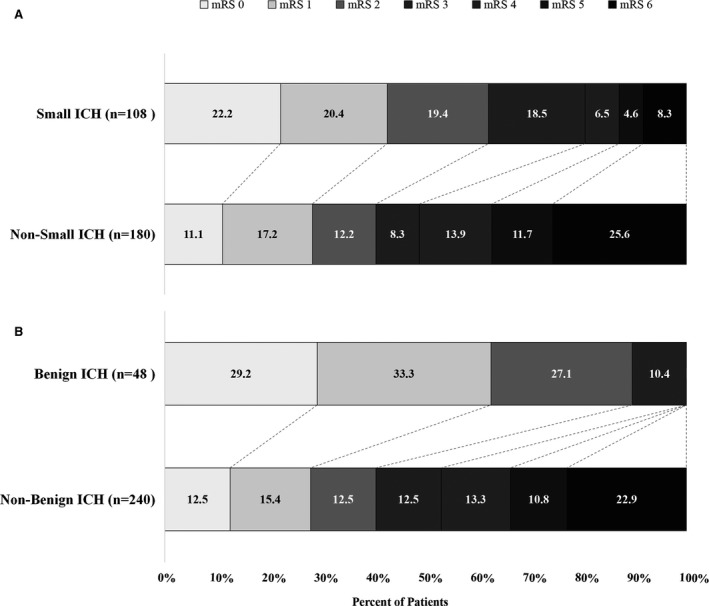
Illustration of modified Rankin Scale (mRS) scores in patients with small intracerebral hemorrhage (ICH) and benign ICH. The percentage of participants with the mRS score obtained at 90 days is shown in each cell. **A**, Distribution of mRS scores in patients with and without small ICH. **B**, Distribution of mRS scores in patients with and without benign ICH. None of the patients with benign ICH had an mRS score of 4 to 6.

At the 3‐month follow‐up, 140 (48.6%) of the 288 patients with ICH were functionally independent (mRS score, 0–2). The distribution of mRS scores in patients with small and benign ICH and those without small and benign ICH is shown in Figure 3. Patients with small ICH were more likely to be functionally independent than those without small ICH (62.0% versus 40.6%; *P*<0.001; Table 1). Strikingly, none of the patients with benign ICH had a poor outcome at 3 months (mRS score, 4–6). Furthermore, patients with benign ICH were more likely to have functional independence compared with those without benign ICH (89.6% versus 40.4%; *P*<0.001; Table 1).

In univariate analyses, age, admission NIHSS score, admission GCS score, presence of intraventricular hemorrhage, baseline ICH volume, and presence of benign ICH were each independently associated with functional independence (*P*<0.05 for all; Table 3).

**Table 3:** Univariate Analysis of Predictors for Functional Independence (mRS Score, 0–2)

Variables	Odds Ratio	95% CI	*P* Value

Mean age, y^a^	0.97	0.95–0.99	0.001^b^
Alcohol consumption	1.17	0.73–1.86	0.510
Smoking	0.92	0.58–1.45	0.706
Hypertension	0.99	0.60–1.64	0.960
Diabetes mellitus	0.79	0.37–1.69	0.542
Systolic blood pressure, mm Hg^a^	0.99	0.98–1.00	0.066
Diastolic blood pressure, mm Hg^a^	1.00	0.98–1.01	0.536
Admission GCS score^a^	1.27	1.17–1.39	<0.001^b^
Admission NIHSS score^a^	0.91	0.88–0.93	<0.001^b^
Baseline ICH volume, mL^a^	0.96	0.94–0.97	<0.001^b^
IVH at baseline CT	0.28	0.16–0.47	<0.001^b^
SAH at baseline CT	0.66	0.31–1.37	0.263
Time from onset to CT, h^a^	1.15	0.99–1.32	0.062
Benign ICH	12.68	4.85–33.16	<0.001^b^

CT indicates computed tomography; GCS, Glasgow Coma Scale; ICH, intracerebral hemorrhage; IVH, intraventricular hemorrhage; mRS, modified Rankin Scale; NIHSS, National Institutes of Health Stroke Scale; SAH, subarachnoid hemorrhage.

a Per unit change in regressor.

b Indicates *P* value <0.05.

The areas under the curve of small ICH and benign ICH in predicting functional independence at 3 months were 0.601 and 0.637, respectively (Figure S2). The sensitivity, specificity, positive predictive value, and negative predictive value of benign ICH for predicting functional independence at 3 months were 30.7%, 96.6%, 90.0%, and 60.0%, respectively.

## Discussion

Although many groups have studied those at high risk for ICH expansion, few have explicitly focused on the low‐risk expanders. In our study, we found that neuroimaging characteristics can successfully mark a cohort of patients at extremely low risk of expansion and poor outcome. We have operationally labeled this finding benign ICH and found that ≈17% of patients met this definition.

Although many groups have found that smaller hematomas may have lower risk of expansion, there are currently no established criteria for defining small ICH. One study defined this as longest diameter of 1.5 cm, and another defined this as ICH volume of <3 mL.^24^, ^25^ Because of a lack of consensus, in our study, we defined small ICH as a function of both size and location. For example, a 10‐mL occipital lobe hematoma may be less harmful than a pontine or thalamic hematoma of the same size. A recent study suggested that intrahematomal hypodensity on CT predicts hematoma expansion and a combination of intrahematomal hypodensity and an existing hematoma expansion score improves prediction of hematoma expansion in patients with ICH.^26^ As noncontrast CT imaging markers predict hematoma expansion, we then further defined a subgroup of “small” hematomas that are regular in shape and homogeneous in density, with no high‐risk features, as benign ICH. As a result, the label benign ICH incorporates information on size, location, and neuroimaging markers of expansion. The results of our study demonstrate that both small ICH and benign ICH were associated with good functional outcomes. However, benign ICH seemed to be a much stronger predictor of functional independence (mRS score, 0–2) at 3 months than a small ICH in the univariate logistic regression model (odds ratio, 12.68 versus 2.40). Furthermore, our results show that benign ICH is highly specific (96.6%) for predicting functional independence at 3 months. We assert that the prognosis of benign ICH is fairly good, and any treatment targeting important harmful pathophysiological processes in ICH is unlikely to change the outcome in patients with benign ICH.

Our findings may have important clinical implications for future clinical trials. Patients with benign ICH may represent a cohort with no opportunity to benefit from trials aimed at expansion. For example, the ATACH‐II (Antihypertensive Treatment of Acute Cerebral Hemorrhage II) trial of intensive blood pressure reduction, thought to reduce ICH expansion,^27^, ^28^, ^29^, ^30^ found no effect on outcome and also enrolled many patients with small‐volume ICHs.^27^ Many of these may have met benign ICH criteria, and acute blood pressure lowering may have minimal effect on them. It may be that patients with benign ICH should be excluded from future trials of antiexpansion therapies.

Furthermore, the concept of benign ICH may add value to existing prognostic scales. Existing scores, such as the ICH score, are useful tools for stratifying for disease severity and guiding prognosis.^31^, ^32^ However, our finding that patients with benign ICH were less likely to have poor outcome compared with those with the same hematoma size suggests that this neuroimaging marker adds additional valuable prognostic information.

Last, our findings have clinical implications as well. Because patients with benign ICH appear to be relatively stable, interventions such as hemostatic therapy, intensive blood pressure lowering, or antiedema treatment with osmotic agents may offer little value. In limited resource settings, such patients likely have less need for intensive monitoring or serial CT scans. Our findings may, therefore, assist in stratifying care to those who most need it.

There are several limitations in our study. First, this is a single‐center study with a relatively small sample size. Second, the choice for benign ICH volume cutoffs was based on our own clinical experience. Third, our study results were not independently validated in an independent cohort. In our country, warfarin was underused and anticoagulant‐associated bleeding was excluded from our study, which may limit the generalizability of our findings to anticoagulant‐associated ICH. Finally, the exact timing of the follow‐up CT scan was at the discretion of the treating physician and not standardized.

Benign ICH is relatively stable and associated with functional independence. These results may assist in triage and resource use when resources or intensive care bed space is limited. In addition, future trials of antiexpansion therapies may benefit from excluding these patients.

## Author Contributions

Q. Li was responsible for the study concept and design and had full access to all of the data in the study. Q. Li, W.‐S. Yang, Shen, X.‐F. Xie, R. Li, Deng, T.‐T. Yang, F.‐J. Lv, F.‐R. Lv, Wu, Tang, and P. Xie performed acquisition or analysis and interpretation of data. Q. Li drafted the manuscript. Q. Li, Wu, Tang, Goldstein, and P. Xie performed critical revision of the manuscript. W.‐S. Yang performed statistical analysis. Q. Li obtained funding. Q. Li and P. Xie were responsible for the administrative, technical, or material support.

## Sources of Funding

This study was supported by grants from the National Key R&D Program of China (2018YFC1312200 and 2018YFC1312203), the Health and Family Planning Commission of Chongqing (2017MSXM014), the China Association for Science and Technology Young Talent Project (2017QNRC001), and the National Natural Science Foundation of China (81200899).

## Disclosures

Goldstein has received consulting and research contracts from CSL Behring and Boehringer Ingelheim. The remaining authors have no disclosures to report.

## Supporting information


**Figure S1.** Cohort selection flowchart.
**Figure S2.** Comparison of receiver‐operating characteristic (ROC) curves in predicting functional independence at 3 months.

## References

[1] Qureshi AI , Mendelow AD , Hanley DF . Intracerebral haemorrhage. Lancet. 2009;373:1632–1644. 19427958 10.1016/S0140-6736(09)60371-8 PMC3138486

[2] van Asch CJ , Luitse MJ , Rinkel GJ , van der Tweel I , Algra A , Klijn CJ . Incidence, case fatality, and functional outcome of intracerebral haemorrhage over time, according to age, sex, and ethnic origin: a systematic review and meta‐analysis. Lancet Neurol. 2010;9:167–176. 20056489 10.1016/S1474-4422(09)70340-0

[3] Zia E , Engström G , Svensson PJ , Norrving B , Pessah‐Rasmussen H . Three‐year survival and stroke recurrence rates in patients with primary intracerebral hemorrhage. Stroke. 2009;40:3567–3573. 19729603 10.1161/STROKEAHA.109.556324

[4] Broderick JP , Diringer MN , Hill MD , Brun NC , Mayer SA , Steiner T , Skolnick BE , Davis SM ; Recombinant Activated Factor VII Intracerebral Hemorrhage Trial Investigators . Determinants of intracerebral hemorrhage growth: an exploratory analysis. Stroke. 2007;38:1072–1075. 17290026 10.1161/01.STR.0000258078.35316.30

[5] Brouwers HB , Greenberg SM . Hematoma expansion following acute intracerebral hemorrhage. Cerebrovasc Dis. 2013;35:195–201. 23466430 10.1159/000346599 PMC3743539

[6] Davis SM , Broderick J , Hennerici M , Brun NC , Diringer MN , Mayer SA , Begtrup K , Steiner T ; Recombinant Activated Factor VII Intracerebral Hemorrhage Trial Investigators . Hematoma growth is a determinant of mortality and poor outcome after intracerebral hemorrhage. Neurology. 2006;66:1175–1181. 16636233 10.1212/01.wnl.0000208408.98482.99

[7] Delcourt C , Huang Y , Arima H , Chalmers J , Davis SM , Heeley EL , Wang J , Parsons MW , Liu G , Anderson CS ; INTERACT1 Investigators . Hematoma growth and outcomes in intracerebral hemorrhage: the INTERACT1 study. Neurology. 2012;79:314–319. 22744655 10.1212/WNL.0b013e318260cbba

[8] Brouwers HB , Chang Y , Falcone GJ , Cai X , Ayres AM , Battey TW , Vashkevich A , McNamara KA , Valant V , Schwab K . Predicting hematoma expansion after primary intracerebral hemorrhage. JAMA Neurol. 2014;71:158–164. 24366060 10.1001/jamaneurol.2013.5433 PMC4131760

[9] Hemphill JC III , Bonovich DC , Besmertis L , Manley GT , Johnston SC . The ICH score: a simple, reliable grading scale for intracerebral hemorrhage. Stroke. 2001;32:891–897. 11283388 10.1161/01.str.32.4.891

[10] Rodriguez‐Luna D , Coscojuela P , Rubiera M , Hill MD , Dowlatshahi D , Aviv RI , Silva Y , Dzialowski I , Lum C , Czlonkowska A , Boulanger JM , Kase CS , Gubitz G , Bhatia R , Padma V , Roy J , Tomasello A , Demchuk AM , Molina CA ; PREDICT/Sunnybrook ICH CTA Study Group . Ultraearly hematoma growth in active intracerebral hemorrhage. Neurology. 2016;87:357–364. 27343067 10.1212/WNL.0000000000002897 PMC4977111

[11] Sato S , Arima H , Hirakawa Y , Heeley E , Delcourt C , Beer R , Li Y , Zhang J , Jüettler E , Wang J , Lavados PM , Robinson T , Lindley RI , Chalmers J , Anderson CS ; INTERACT Investigators . The speed of ultraearly hematoma growth in acute intracerebral hemorrhage. Neurology. 2014;83:2232–2238. 25378675 10.1212/WNL.0000000000001076 PMC4277674

[12] Rodriguez‐Luna D , Rubiera M , Ribo M , Coscojuela P , Piñeiro S , Pagola J , Hernandez‐Guillamon M , Ibarra B , Romero F , Alvarez‐Sabin J , Montaner J , Molina CA . Ultraearly hematoma growth predicts poor outcome after acute intracerebral hemorrhage. Neurology. 2011;77:1599–1604. 21998314 10.1212/WNL.0b013e3182343387

[13] Boulouis G , Morotti A , Brouwers HB , Charidimou A , Jessel MJ , Auriel E , Pontes‐Neto O , Ayres A , Vashkevich A , Schwab KM , Rosand J , Viswanathan A , Gurol ME , Greenberg SM , Goldstein JN . Association between hypodensities detected by computed tomography and hematoma expansion in patients with intracerebral hemorrhage. JAMA Neurol. 2016;73:961–968. 27323314 10.1001/jamaneurol.2016.1218 PMC5584601

[14] Li Q , Zhang G , Huang YJ , Dong MX , Lv FJ , Wei X , Chen JJ , Zhang LJ , Qin XY , Xie P . Blend sign on computed tomography: novel and reliable predictor for early hematoma growth in patients with intracerebral hemorrhage. Stroke. 2015;46:2119–2123. 26089330 10.1161/STROKEAHA.115.009185

[15] Li Q , Zhang G , Xiong X , Wang XC , Yang WS , Li KW , Wei X , Xie P . Black hole sign: novel imaging marker that predicts hematoma growth in patients with intracerebral hemorrhage. Stroke. 2016;47:1777–1781. 27174523 10.1161/STROKEAHA.116.013186

[16] Li Q , Liu QJ , Yang WS , Wang XC , Zhao LB , Xiong X , Li R , Cao D , Zhu D , Wei X , Xie P . Island sign: an imaging predictor for early hematoma expansion and poor outcome in patients with intracerebral hemorrhage. Stroke. 2017;48:3019–3025. 29018128 10.1161/STROKEAHA.117.017985

[17] Boulouis G , Morotti A , Brouwers HB , Charidimou A , Jessel MJ , Auriel E , Pontes‐Neto O , Ayres A , Vashkevich A . Noncontrast computed tomography hypodensities predict poor outcome in intracerebral hemorrhage patients. Stroke. 2016;47:2511–2516. 27601380 10.1161/STROKEAHA.116.014425 PMC5039101

[18] Li Q , Yang WS , Wang XC , Cao D , Zhu D , Lv FJ , Liu Y , Yuan L , Zhang G , Xiong X , Li R , Hu YX , Qin XY , Xie P . Blend sign predicts poor outcome in patients with intracerebral hemorrhage. PLoS One. 2017;12:e0183082. 28829797 10.1371/journal.pone.0183082 PMC5568736

[19] Shimoda Y , Ohtomo S , Arai H , Okada K , Tominaga T . Satellite sign: a poor outcome predictor in intracerebral hemorrhage. Cerebrovasc Dis. 2017;44:105–112. 28605739 10.1159/000477179

[20] Anderson CS , Huang Y , Wang JG , Arima H , Neal B , Peng B , Heeley E , Skulina C , Parsons MW , Kim JS , Tao QL ; INTERACT Investigators . Intensive blood pressure reduction in acute cerebral haemorrhage trial (INTERACT): a randomised pilot trial. Lancet Neurol. 2008;7:391–399. 18396107 10.1016/S1474-4422(08)70069-3

[21] Anderson CS , Heeley E , Huang Y , Wang J , Stapf C , Delcourt C , Lindley R , Robinson T , Lavados P , Neal B ; INTERACT2 Investigators . Rapid blood‐pressure lowering in patients with acute intracerebral hemorrhage. N Engl J Med. 2013;368:2355–2365. 23713578 10.1056/NEJMoa1214609

[22] Kothari RU , Brott T , Broderick JP , Barsan WG , Sauerbeck LR , Zuccarello M , Khoury J . The ABCs of measuring intracerebral hemorrhage volumes. Stroke. 1996;27:1304–1305. 8711791 10.1161/01.str.27.8.1304

[23] Dowlatshahi D , Demchuk AM , Flaherty ML , Ali M , Lyden PL , Smith EE ; VISTA Collaboration . Defining hematoma expansion in intracerebral hemorrhage: relationship with patient outcomes. Neurology. 2011;76:1238–1244. 21346218 10.1212/WNL.0b013e3182143317 PMC3068004

[24] Kim JS , Lee JH , Lee MC . Small primary intracerebral hemorrhage: clinical presentation of 28 cases. Stroke. 1994;25:1500–1506. 8023369 10.1161/01.str.25.7.1500

[25] Dowlatshahi D , Yogendrakumar V , Aviv RI , Rodriguez‐Luna D , Molina CA , Silva Y , Dzialowski I , Czlonkowska A , Boulanger JM , Lum C , Gubitz G , Padma V , Roy J , Kase CS , Bhatia R , Hill MD , Demchuk AM ; PREDICT/Sunnybrook ICH CTA Study Group . Small intracerebral hemorrhages have a low spot sign prevalence and are less likely to expand. Int J Stroke. 2016;11:191–197. 26783310 10.1177/1747493015616635

[26] VanDerWerf J , Kurowski D , Siegler J , Ganguly T , Cucchiara B . Combination of intra‐hematomal hypodensity on CT and BRAIN scoring improves prediction of hemorrhage expansion in ICH. Neurocrit Care. 2018;29:40–46. 29411303 10.1007/s12028-018-0507-y

[27] Qureshi AI , Palesch YY , Barsan WG , Hanley DF , Hsu CY , Martin RL , Moy CS , Silbergleit R , Steiner T , Suarez JI ; ATACH‐2 Trial Investigators and the Neurological Emergency Treatment Trials Network . Intensive blood‐pressure lowering in patients with acute cerebral hemorrhage. N Engl J Med. 2016;375:1033–1043. 27276234 10.1056/NEJMoa1603460 PMC5345109

[28] Butcher K , Selim M . Acute blood pressure management in intracerebral hemorrhage: equipoise resists an attack. Stroke. 2016;47:3065–3066. 27895301 10.1161/STROKEAHA.116.015060 PMC5134899

[29] Morotti A , Brouwers HB , Romero JM , Jessel MJ , Vashkevich A , Schwab K , Afzal MR , Cassarly C , Greenberg SM , Martin RH , Qureshi AI , Rosand J , Goldstein JN ; Antihypertensive Treatment of Acute Cerebral Hemorrhage II and Neurological Emergencies Treatment Trials Investigators . Intensive blood pressure reduction and spot sign in intracerebral hemorrhage: a secondary analysis of a randomized clinical trial. JAMA Neurol. 2017;74:950–960. 28628707 10.1001/jamaneurol.2017.1014 PMC5584592

[30] Morotti A , Boulouis G , Romero JM , Brouwers HB , Jessel MJ , Vashkevich A , Schwab K , Afzal MR , Cassarly C ; ATACH‐II and NETT Investigators . Blood pressure reduction and noncontrast CT markers of intracerebral hemorrhage expansion. Neurology. 2017;89:548–554. 28701501 10.1212/WNL.0000000000004210 PMC5562954

[31] Hemphill JC III , Farrant M , Neill TA Jr . Prospective validation of the ICH score for 12‐month functional outcome. Neurology. 2009;73:1088–1094. 19726752 10.1212/WNL.0b013e3181b8b332 PMC2764394

[32] Cheung RT , Zou LY . Use of the original, modified, or new intracerebral hemorrhage score to predict mortality and morbidity after intracerebral hemorrhage. Stroke. 2003;34:1717–1722. 12805488 10.1161/01.STR.0000078657.22835.B9

