# Supplementary material for: Benign Intracerebral Hemorrhage: A Population at Low Risk for Hematoma Growth and Poor Outcome
Source: J Am Heart Assoc. 2019 Apr 11;8(8):e011892. doi: 10.1161/JAHA.118.011892 (PMC6507215; doi:10.1161/JAHA.118.011892)

SUPPLEMENTAL MATERIAL

Figure S1. Cohort Selection Flowchart.

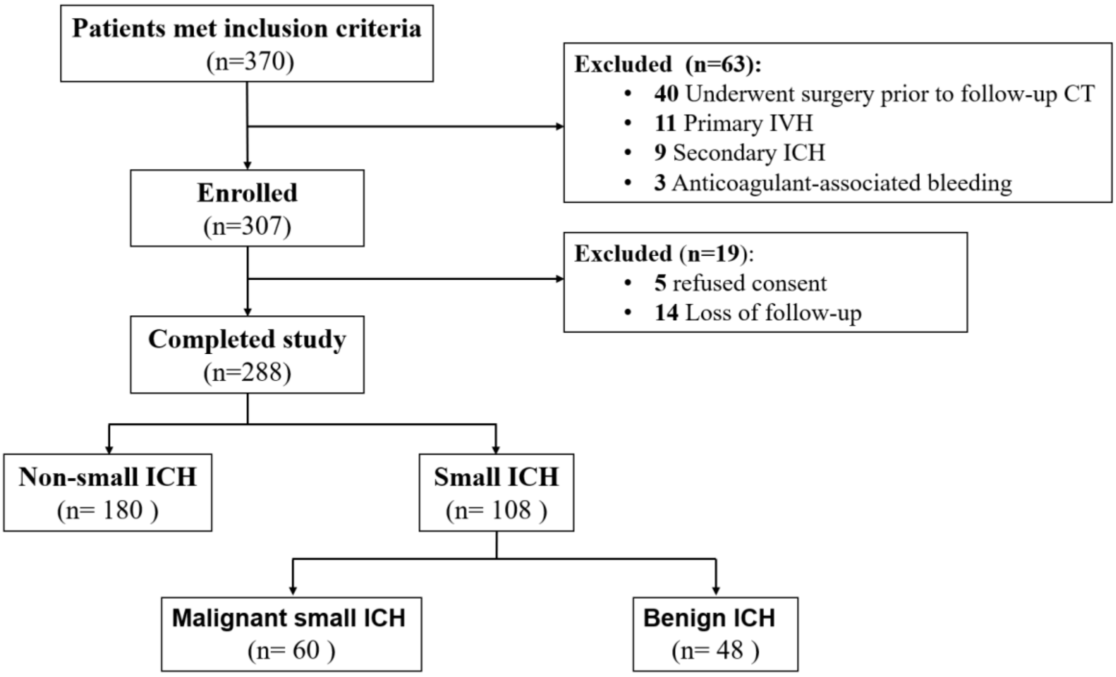

Figure S2. Comparison of Receiver-operating characteristic (ROC) curves in predicting functional independence at 3 months.

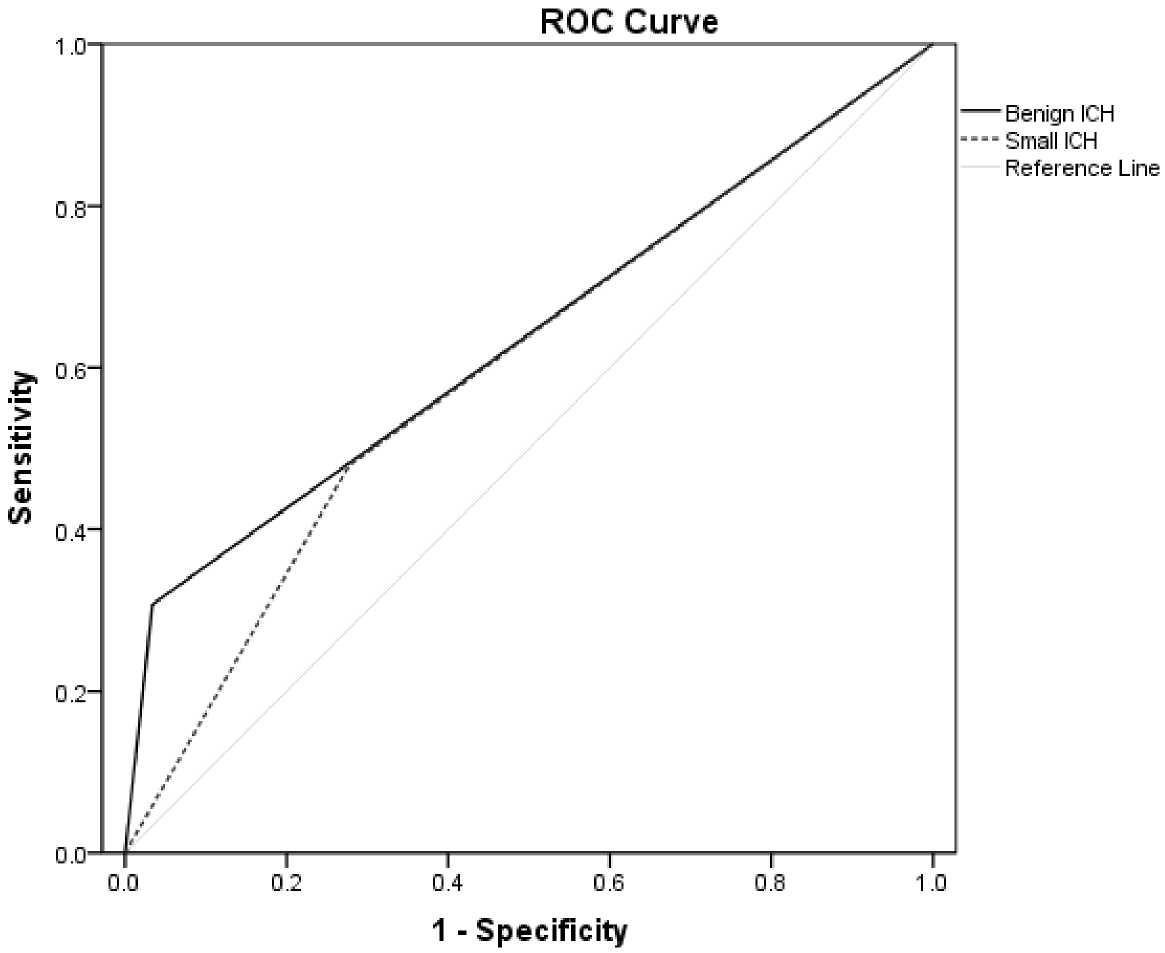

Supplement: Supplementary file 1 — Figure S1. Cohort selection flowchart. Figure S2. Comparison of receiver‐operating characteristic (ROC) curves in predicting functional independence at 3 months. [file JAH3-8-e011892-s001.pdf]
